# Intraocular Pressure Control after Implantation of an Ahmed Glaucoma Valve in Eyes with a Failed Trabeculectomy

**DOI:** 10.5005/jp-journals-10008-1209

**Published:** 2016-10-29

**Authors:** Rui B Schimiti, Ricardo Y Abe, Carla M Tavares, Jose PC Vasconcellos, Vital P Costa

**Affiliations:** 1 Professor and Assistant, Department of Ophthalmology, Pontifical University; Eye Hospital of Londrina (HOFTALON), Londrina, PR, Brazil University of Campinas, São Paulo, Brazil; 2 Postgraduate Student, Department of Ophthalmology, University of Campinas, São Paulo, Brazil; 3 Resident, Department of Ophthalmology, University of Campinas, São Paulo, Brazil; 4 Professor, Department of Ophthalmology, University of Campinas, São Paulo, Brazil; 5 Professor, Department of Ophthalmology, University of Campinas, São Paulo, Brazil

**Keywords:** Ahmed glaucoma valve, Glaucoma surgery, Intraocular pressure, Retrospective study.

## Abstract

**Aim:**

To evaluate the results of Ahmed glaucoma valve (AGV) in eyes with a failed trabeculectomy.

**Materials and methods:**

This retrospective study evaluated 61 eyes with a failed trabeculectomy that underwent implantation of an AGV due to uncontrolled intraocular pressure (IOP) on maximal medical therapy. Success was defined as IOP ≤ 21 mm Hg (criterion 1) or 20% reduction in IOP (criterion 2) with or without antiglaucoma medications. Persistent hypotony, loss of light perception, and reoperation for IOP control were defined as failure.

**Results:**

Mean preoperative IOP and mean lOPs at 6, 12, and 24 months were 21.93 ± 6.32 mm Hg (n = 61), 14.15 ± 4.33 mm Hg (n = 59), 13.21 ± 4.44 mm Hg (n = 56), and 13.60 ± 3.27 mm Hg (n = 25) respectively. Mean number of antiglaucoma medications preoperatively and at 6, 12, and 24 months was 3.95 ± 0.85, 2.19 ± 1.38, 2.48 ± 1.44, and 2.40 ± 1.32 respectively. The reductions in the number of medications and IOP measurements were statistically significant at all time intervals (p < 0.001, Wilcoxon signed rank test). According to criterion 1, the Kaplan-Meier survival curve disclosed success rates of 75% at 12 and 24 months. According to criterion 2, the success rates were 57% at 12 months and 55% at 24 months. The most frequent complications were hypertensive phase (18%) and shallow anterior chamber (16.4%).

**Conclusion:**

The AGV may effectively reduce IOP in eyes that had a failed trabeculectomy.

**Clinical significance:**

The AGV is an alternative in eyes with a failed trabeculectomy.

**How to cite this article:**

Schimiti RB, Abe RY, Tavares CM, Vasconcellos JPC, Costa VP. Intraocular Pressure Control after Implantation of an Ahmed Glaucoma Valve in Eyes with a Failed Trabeculectomy. J Curr Glaucoma Pract 2016;10(3):97-103.

## INTRODUCTION

The need to perform multiple and sequenced surgical procedures is a relatively common situation in refractory glaucoma. Although trabeculectomy with mitomycin C is still the most popular surgical procedure, the number of glaucoma implant surgeries has increased in recent years, possibly because they provide filtering blebs closer to the equator, with benefits, such as lower risk of late endophthalmitis and good long-term intraocular pressure (IOP) control.^1-3^ The Baerveldt Glaucoma Implant (AMO Inc, Irvine, CA, USA) (BGI) and the Ahmed glaucoma valve (AGV; New World Medical, Inc., Rancho Cucamonga, CA, USA) are the most common types of devices used worldwide, and comparative studies have demonstrated a lower incidence of severe complications, but greater dependence on antiglaucomatous medication for adequate IOP control with the latter.^4-6^

Previous studies evaluating the performance of AGV included a very heterogeneous population of patients or groups with various types of secondary glaucomas, such as neovascular, inflammatory, postkeratoplasty, or pediatric glaucoma.^7-16^ Studies that specifically measured the performance of this procedure in adults with primary open angle glaucoma (POAG) and primary angle closure glaucoma (PACG) that had a failed trabeculectomy were not found in the literature. The efficacy and safety of the AGV in this situation are not well established, and this information would be valuable when making surgical decisions. Therefore, we conducted a study to evaluate the IOP control, the most common complications, and the risk factors related to surgical failure in eyes that had one or more failed trabeculectomies and required an AGV implantation.

## MATERIALS AND METHODS

### Patient Selection

This was a retrospective study evaluating patients with one or more failed trabeculectomies who underwent implantation of an AGV (models S2 and FP7) due to uncontrolled IOP under maximal tolerated medical therapy. Only patients with at least 1 year of follow-up were included in the study, unless failure occurred before that period. All procedures were performed between January 2000 and November 2012. This study was approved by the Ethics Committee of the University of Campinas and adhered to the tenets of the Declaration of Helsinki. Medical records were retrospectively reviewed for preoperative and postoperative follow-up information. At each visit during follow-up, subjects underwent a comprehensive ophthalmic examination, including Snellen best corrected visual acuity, slit-lamp biomicros-copy, Goldmann applanation tonometry, gonioscopy, and dilated fundoscopy examination using a 78-diopter lens.

### Outcome Measures

Outcome measures included IOP, visual acuity, number of antiglaucoma medications, surgical success, and complications. Surgical success was defined as (a) criterion 1: IOP ≤ 21 mm Hg with or without antiglaucoma medications; and (b) criterion 2: 20% reduction of preoperative IOP with or without antiglaucoma medications.

Persistent hypotony (IOP ≤ 5 mm Hg in at least two consecutive visits after 3 months of follow-up), loss of light perception, and reoperation for IOP control were also defined as failure. Flat anterior chambers were graded according to the classification proposed by Spaeth.^17^ An early hypertensive phase (EHP) was defined as an IOP greater than 21 mm Hg within the first 2 months after surgery and after a reduction of IOP to less than 21 mm Hg had been achieved during the first postoperative week.

Early postoperative complications were defined as those developing within 30 days after the procedure, and late postoperative complications were defined as those that occurred after this period. Reoperation was defined as any additional surgery requiring a return to the operating room and was subdivided in reoperation for complication and reoperation for IOP control.

We also evaluated the number of eyes that showed a significant visual acuity loss, defined as a reduction of two or more Snellen lines or category change from baseline (i.e., counting fingers to hand motion).

### Surgical Technique

Surgeries were performed by one of three experienced attending glaucoma surgeons (JPV, RBC, VPC) or glaucoma fellows under their direct surgical supervision. The AGV models used were the S2 (184 mm^2^ surface area) and FP7 (184 mm^2^ surface area). All surgeries were performed with peribulbar anesthesia. A fornix-based conjunctival flap was fashioned in the superotemporal, superonasal, or inferonasal quadrants. Ahmed glaucoma valve was primed by flushing balanced salt solution through the tube to confirm patency. The anterior edge of the plate was secured with 9-0 nylon or 8-0 silk sutures to the sclera at least 8 mm from the limbus. A 23-gauge needle was used to enter the anterior chamber 1 mm posterior to the limbus, and viscoelastic (methylcellulose 2%) was injected before tube insertion. A rectangular donor scleral patch graft (4 × 6 mm) was fashioned and sutured over the tube using 10-0 nylon sutures. The conjunctiva was also secured with 10-0 nylon sutures. No antimetabolites were used in the procedure. Follow-up visits were scheduled after 1 day; 3 days; 1 week; 2 weeks; 1, 3, 6, 12, 18, and 24 months. All patients received a standard regimen of topical antibiotic drops (moxifloxacin hydrochloride) qid, discontinued after 2 weeks. Topical corticosteroids drops (prednisolone acetate ophthalmic suspension 1%) were used initially six times daily and tapered gradually over 8 to 10 weeks depending on the degree of inflammation. Glaucoma medications were prescribed according to IOP measurements and the severity of the disease.

### Statistical Analysis

Preoperative and postoperative IOP measurements and number of medications were compared using the Wilcoxon signed rank test. Cumulative survival rates were calculated using Kaplan-Meier survival analysis. Cox proportional Hazards regression analysis with forward stepwise elimination was used to assess potential predictors for surgical failure. We included variables previously listed as risk factors according to the litera-ture.^5,6^ The following variables were studied: Race, eye (right or left), glaucoma diagnosis, gender, preoperative IOP, previous glaucoma surgeries, preoperative glaucoma medications, preoperative use of oral acetazolamide, interval between last surgery and implant, type of implant, quadrant of valve implantation, and occurrence of hypertensive phase in the postoperative period. Data analyses were performed using Statistical Package for the Social Sciences (SPSS) version 19.0 (Inc., Chicago, IL). All reported probability values are two-tailed, and p < 0.05 was considered statistically significant.

## RESULTS

A total of 61 eyes from 61 patients with one or more failed trabeculectomies who underwent implantation of an AGV were enrolled in the study. Table 1 shows the clinical and demographic characteristics of the subjects. Among the patients, 43 (70.5%) were Caucasian and 18 (29.5%) were African-American; 55 patients (90.2%) had POAG, and 6 patients (9.8%) had PACG. Patients were followed for an average of 17.28 ± 6.24 months (3 to 24 months). Mean age was 68.1 ± 11.8 years. Among the 61 eyes, 29 (47.5%) received the Ahmed S2 valve, and 32 eyes (52.5%) received the Ahmed FP7 valve. Mean number of previous surgeries was 1.92 ± 0.23. The mean interval between the last surgical procedure and the implantation of the AGV was 38.15 ± 5.73 months. In this series, 17 (27.9%) eyes were pseudophakic, and 44 (72.1%) eyes were phakic. Mean IOP decreased from 21.93 ± 6.32 mm Hg prior to surgery to 13.60 ± 3.27 mm Hg at 2 years of follow-up (Table 2). Mean number of medications decreased from 3.95 ± 0.85 at baseline to 2.40 ± 1.32 at 2 years of follow-up (Table 3). The reductions in the number of medications and IOP measurements were statistically significant at all time intervals (p < 0.001, Wilcoxon signed rank test).

**Table 1:** **Table 1:** Clinical and demographic characteristics

*Variable*		*Patients (n = 61)*	
Age		68.1 ± 11.8 years	
Sex		Female = 21 (34.4%)	
		Male = 40 (65.6%)	
Eye		Right = 32 (52.5%)	
		Left = 29 (47.5%)	
Race		Caucasian 43 (70.5%)	
		African-American 18 (29.5%)	
Type of glaucoma		POAG = 55 (90.2%)	
		PACG = 6 (9.8%)	
Type of implant (model)		S2 = 29 (47.5%)	
		FP7 = 32 (52.5%)	
Mean follow-up		17.28 ± 6.24 months	
Interval between last surgery and implant (missing eight patients - unavailable)		38.15 ± 5.73 months	

**Table 2:** **Table 2:** Mean IOP at different intervals

*Interval*		*n*		*Mean IOP (mm Hg)*		*Std. Deviation*	
Pre-op		61		21.93		6.32	
1 day		61		9.02		4.72	
7 days		60		9.82		4.54	
14 days		59		13.46		5.86	
1 month		60		15.67		5.28	
3 months		59		13.88		5.35	
6 months		59		14.15		4.33	
12 months		56		13.21		4.44	
18 months		34		12.21		3.37	
24 months		25		13.60		3.27	

**Table 3:** **Table 3:** Mean number of antiglaucoma medications at different intervals

*Interval*		*n*		*Mean*		*Std. Deviation*	
Pre-op		61		3.95		0.85	
7 days		60		0.05		0.22	
14 days		59		0.22		0.65	
30 days		60		0.80		0.99	
3 months		59		1.59		1.19	
6 months		59		2.19		1.38	
12 months		56		2.48		1.44	
18 months		34		2.24		1.37	
24 months		25		2.40		1.32	

**Graphs 1A and B:**
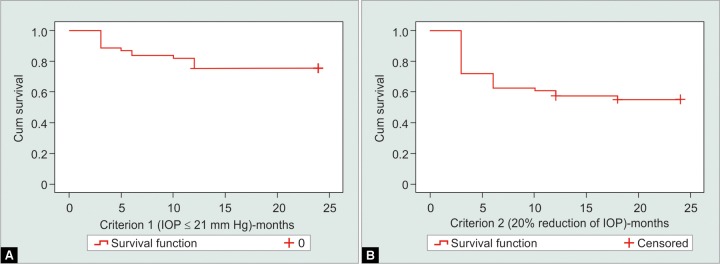
Kaplan-Meier survival curves according to criterion 1 (A) and criterion 2 (B)

Kaplan-Meier survival curves according to criterion 1 (IOP ≤ 21 mm Hg) and criterion 2 (IOP reduction > 20% from baseline IOP) are shown in Graphs 1A and B. Utilizing criterion 1, the analysis showed cumulative survival rates of 84, 75, and 75% after 6, 12, and 24 months respectively. Utilizing criterion 2, the analysis showed cumulative survival rates of 62, 57, and 55% after 6, 12, and 24 months respectively. Table 4 shows the reasons for surgical failure. The most common reason for failure was inadequate IOP control. We found 20 patients with early complications: Hypertensive phase (18%), which occurred 23 days (mean) after surgery; shallow anterior chamber (16.4%); choroidal detachment (4.9%); conjunctival leakage (1.6%); and hyphema (1.6%). Regarding late postoperative complications, we had 8 patients: Cystoid macular edema or macular hole (4.9%); tube exposure (3.3%); phthisis bulbi (3.3%); plate exposure (1.6%), and endophthalmi-tis (1.6%). We did not observe a single case of early or late postoperative hypotony. We had a total of 9 (14.7%) patients who underwent reoperation for complications: Anterior chamber paracentesis with viscoelastic (8.2%); scleral patch (3.3%); tube reposition (1.6%); vitrectomy (1.6%); and device explantation (1.6%).

**Table 4:** **Table 4:** Reasons for surgical failure

Additional glaucoma		Second tube (n = 3)	
surgery		Cyclophotocoagulation (n = 8)	
		Argon laser trabeculoplasty (n = 1)	
Pressure > 21 mm Hg		n = 9	
Pressure reduction < 20%		n = 21	
of presurgical IOP			
Device explantation		n = 1	
Loss of light perception		n = 1	

In univariate and multivariate Cox models, considering both success criterion, Caucasian race was found to be associated with a lower risk for treatment failure (p < 0.001 in both models). All the other variables were not associated with treatment failure.

Baseline, 12 and 24 months average postoperative logMAR visual acuities were 0.92 ± 0.73, 0.99 ± 0.81, and 0.90 ± 0.85 respectively. Ten (16.4%) eyes showed significant visual acuity loss from baseline.

## DISCUSSION

Different types of glaucoma drainage devices have been increasingly used in the treatment of refractory glaucoma and after previously failed filtering procedures.^18-20^ The unidirectional flow-restricted AGV and the open tube BGI are the most commonly used glaucoma drainage devices in the United States.^4,6^ The Ahmed Baerveldt Comparison (ABC) study and the Ahmed versus Baerveldt Study were multicenter prospective clinical trials comparing these two devices.^4-6^ Both studies concluded that the BGI produced greater IOP reduction, requiring fewer adjunctive medications and glaucoma reoperations compared with the AGV after 5 and 3 years of follow-up respectively, but the AGV showed a significantly lower number of complications.

The use of shunt devices after failed trabeculectomies has been reinforced by the results of the Tube versus Trabeculectomy Study (TVT), which suggested that the implantation of a Baerveldt device is safe and effective in eyes that had previous intraocular surgery (i.e., phacoemulsification or trabeculectomy).^21,22^ However, there are no data from the TVT indicating the success rates in the subgroup of patients who had previously failed trabecu-lectomies, which could be compared with our results. Furthermore, the TVT included the use of the BGI, whose results are different from those obtained with the AGV.

A systematic review and meta-analysis that evaluated six studies comparing trabeculectomy and the AGV concluded that the latter was equivalent to trabeculectomy in reducing IOP and number of medications, presenting the same success rates, but with a lower incidence of adverse effects.^23^ Notwithstanding, this review compared studies investigating a heterogeneous group of patients, including primary surgeries, eyes with neovascular glaucoma, aphakic glaucoma, and postcyclophotocoagulation. In fact, the vast majority of studies evaluating the performance of drainage implants involve a mix of refractory cases, with few studies evaluating a homogeneous group of eyes with lower risk for surgical failure.^5,22,24-27^

Our series evaluated the effectiveness of the AGV to control the IOP in patients with POAG (90.2%) and PACG (9.8%) who had one or more prior failed trabeculectomies, followed for an average of 17.28 ± 6.24 months. After 12 and 24 months of follow-up, our study showed a decrease in IOP of 37.3 ± 19.8% and 38.7 ± 18.7% respectively. Wilson et al,^27^ in a study investigating the performance of the AGV (59 eyes) in individuals with POAG and PACG and no prior intraocular surgery, found a 35.9 and 41.3% IOP reduction after 12 and 24 months of follow-up. Tran et al^25^ evaluated eyes with open-angle glaucoma with or without previous intraocular surgery undergoing AGV implantation and observed IOP reductions of 36.1 and 41.2% after 12 and 24 months respectively. Other studies evaluating the IOP after AGV implantation in different types of refractory glaucoma showed IOP reductions ranging from 46.1 to 71.3% with different follow-up periods.^11,28-30^ Although our study did not include patients with secondary glaucoma, it presented decreases in IOP similar to those found in other studies, when considering the same follow-up.

Our study also demonstrated significant reductions in the mean number of medications relative to baseline, ranging from 32.3 ± 41.6 to 28.3 ± 47.1% at 12 and 24 months respectively. These results tend to be worse than those reported in previously published studies, which varied from 38.2 to 66.6% at 12 months, and from 42.9 to 45.5% at 24 months.^5,25,26^ This discrepant result may be secondary to the high percentage of African-American patients in our cohort.

Different studies used different success criteria, which makes it very difficult to establish any comparison. In addition, previous studies involved eyes with or without previous surgery, a single or multiple surgeons, which make comparisons even more difficult. Nevertheless, alternative failure criteria, such as the presence of ocular hypotony (IOP ≤5 mm Hg), loss of light perception, additional procedures for IOP control (e.g., cyclodestructive procedures), or removal of the implant were uniformly adopted by different studies.^11,25,27-30^ Wilson et al^27^ compared the one-surgeon probability of success after the implantation of the S2 AGV in a group of eyes with no prior intraocular surgery and defined success as IOP of less than 21 mm Hg and at least 15% reduction from preoperative IOP. After 12 and 24 months, the success rates were 93.32 and 84.74% respectively. In their study evaluating the AGV in eyes with POAG operated by three surgeons, Tran et al^25^ adopted four different criteria to define success. When the criterion of IOP ≤21 mm Hg and an at least reduction of IOP ≥15% was considered, the Kaplan-Meier cumulative probability of success depicted levels of 81 and 64% at 12 and 24 months respectively.

The ABC study compared the effectiveness of the AGV and the BGI in individuals with previous intraocular surgery or refractory glaucoma (performed by different surgeons). Defining failure as IOP > 21 mm Hg or less than 20% reduction below baseline, the ABC study showed in the Ahmed group cumulative survival rates of 78.8, 70.8, and 44.7% after 12, 24, and 60 months respectively.^5^ In a retrospective study, Tsai et al^26^ compared the same two implants in a group of refractory glaucoma patients, adopting the following criteria for surgical failure: An IOP > 21 or <6 mm Hg. They found a cumulative probability of success for the Ahmed group of 72.2, 66, and 62% at 12, 24, and 48 months respectively.

In our retrospective, multiple surgeon study, the cumulative probabilities of success according to criterion 1 IOP ≤ 21 mm Hg) were 84, 75, and 75% after 6, 12, and 24 months respectively. According to criterion 2 (IOP reduction ≥20% from baseline IOP), the analysis showed cumulative survival rates of 62, 57, and 55% after 6, 12, and 24 months respectively. The most common reason for failure was the inability to reach 20% reduction from baseline IOP.

In our series, early postoperative complications occurred in 20 (32.8%) patients. The most frequent early complication was the hypertensive phase, which occurred in 11 eyes (18%) after an average of 23 days, followed by shallow anterior chamber (16.4%) and choroidal detachment (4.9%). Probably due to the restriction mechanism present in the AGV, the early complications seem to be less frequent with this device, although the IOP levels are higher when compared with the BGI or trabeculec-tomy.^21,31^ In different studies, the occurrence of shallow anterior chamber after AGV implantation ranged from 10.6 to 19% and the presence of choroidal detachment ranged from 12 to 15%.^6,25,27,31,32^ Our surgical technique tends to locate the tube closer to the iris plane, filling the anterior chamber with 2% methylcellulose viscoelastic, allowing the anterior chamber to remain more stable in the early postoperative days, and reducing the occurrence of corneal edema. This also avoids the abrupt decompression of the anterior chamber, theoretically reducing the occurrence of choroidal detachment and suprachoroidal hemorrhage. Nevertheless, there was a 9.8% incidence of shallow anterior chamber grades 1 and 2, and 6.6% of shallow anterior chamber grade 3, the latter requiring paracentesis and viscoelastic injection in the anterior chamber. We had eight (13.1%) patients with late complications. These numbers compared favorably with those of the TVT study,^21^ where 37 and 18% of the patients who underwent a trabeculectomy showed early and late complications respectively.

Devgan et al^33^ observed that African-American individuals more often require surgical intervention than white patients, whereas other authors have demonstrated that they present a higher risk for failure after glaucoma surgery when compared with Caucasians.^34,35^ This can be justified by the greater fibrovascular proliferation and a more intense healing process in these eyes, even when using antiproliferative agents in the intraoperative period.^36^ A retrospective case-control study compared the performance of the AGV in 86 eyes of Caucasians (n = 43) and African-Americans followed for 2.3 and 2.5 years respectively. The authors found a higher risk of surgical failure in African-Americans (91 and 75% of success at 1 and 3 years respectively) compared with the group of Caucasians (100 and 96% of success at 1 and 3 years respectively).^37^ In the present study, considering both success criteria, the multivariate analysis showed that Caucasian race had a lower risk of implant failure.

The occurrence of a hypertensive phase has been associated with an increased risk of surgical failure following AGV implantation due to the fibrosis surrounding the plate of the drainage device, causing an increase in the resistance to the flowing of aqueous humor.^38,39^ In fact, the treatment with aqueous suppressant has been suggested by two recent prospective studies in the early postoperative period, while IOPs were still in the low-teens to reduce the incidence of IOP spikes associated with the hypertensive phase and improve the success rate.^40,41^ Despite being the most frequent early complication in our study, hypertensive phase was not a risk factor for surgical failure, both in the univariate and multivariate analyses.

The present study has limitations due to its retrospective nature. One of the factors to be considered is the lack of definition of the exact location of previous filtering surgeries due to the large number of cases where the initial procedure was performed in different hospitals and over time. Although, due to iridectomy, the site of trabeculectomy might supposedly be observed, many descriptions of medical records do not show this information. Regarding complications, it is known that many complications are not properly recorded. It is possible that minor unexpected events, such as small leaks, dellen, or hyphema, have not been adequately described. Another important limitation is the one related to survival when surgery was performed with low baseline IOP. It is possible that surgery was indicated, because patients were not tolerating their medications or were not complaining. Hence, their baseline IOP did not reflect the IOP control in real life.

Despite these limitations, this retrospective study showed that AGV implantation after failed trabeculec-tomy provides good IOP control with a reduction in the number of medications and low complication rates. It also confirmed that the expected success rates of this procedure in African-Americans are significantly lower than in Caucasians. Prospective, randomized studies are needed in order to establish the actual effectiveness of this procedure, establishing more precisely its success rates and risk factors.

## CONCLUSION

Ahmed glaucoma valve insertion may effectively reduce IOP in eyes with uncontrolled glaucoma and failed trabeculectomy.

## CLINICAL SIGNIFICANCE

The AGV is an alternative in eyes with a failed trabeculectomy.

## References

[1] Stein JD, Ruiz D Jr, Belsky D, Lee PP, Sloan FA (2008). Longitudinal rates of postoperative adverse outcomes after glaucoma surgery among Medicare beneficiaries 1994 to 2005. Ophthalmology.

[2] Ramulu PY, Corcoran KJ, Corcoran SL, Robin AL (2007). Utilization of various glaucoma surgeries and procedures in Medicare beneficiaries from 1995 to
2004. Ophthalmology.

[3] Lim KS, Allan BD, Lloyd AW, Muir A, Khaw PT (1998). Glaucoma drainage devices; past, present, and future.. Br J Ophthalmol.

[4] Barton K, Feuer WJ, Budenz DL, Schiffman J, Costa VP, Godfrey DG, Buys YM (2014). Ahmed Baerveldt Comparison Study Group. Three-year treatment outcomes in the Ahmed Baerveldt comparison study.. Ophthalmology.

[5] Budenz DL, Barton K, Gedde SJ, Feuer WJ, Schiffman J, Costa VP, Godfrey DG, Buys YM (2015). Ahmed Baerveldt Comparison Study Group. Five-year treatment outcomes in the Ahmed Baerveldt comparison study.. Ophthalmology.

[6] Christakis PG, Tsai JC, Kalenak JW, Zurakowski D, Cantor LB, Kammer JA, Ahmed II (2013). The Ahmed versus Baerveldt study: three-year treatment outcomes.. Ophthalmology.

[7] Arcieri ES, Paula JS, Jorge R, Barella KA, Arcieri RS, Secches DJ, Costa VP (2015). Efficacy and safety of intravitreal bevacizumab in eyes with neovascular glaucoma undergoing Ahmed glaucoma valve implantation: 2-year follow-up.. Acta Ophthalmol.

[8] Ayyala RS, Zurakowski D, Smith JA, Monshizadeh R, Netland PA, Richards DW, Layden WE (1998). A clinical study of the Ahmed glaucoma valve implant in advanced glaucoma.. Ophthalmology.

[9] Chen A, Yu F, Law SK, Giaconi JA, Coleman AL, Caprioli J (2015). Valved glaucoma drainage devices in pediatric glaucoma: retrospective long-term outcomes.. JAMA Ophthalmol.

[10] Chen TC, Bhatia LS, Walton DS (2005). Ahmed valve surgery for refractory pediatric glaucoma: a report of 52 eyes.. J Pediatr Ophthalmol Strabismus.

[11] Im YW, Lym HS, Park CK, Moon JI (2004). Comparison of mitomy-cin C trabeculectomy and Ahmed valve implant surgery for neovascular glaucoma.. J Korean Ophthalmol Soc.

[12] Lai JS, Poon AS, Chua JK, Tham CC, Leung AT, Lam DS (2000). Efficacy and safety of the Ahmed glaucoma valve implant in Chinese eyes with complicated glaucoma.. Br J Ophthalmol.

[13] Panda A, Prakash VJ, Dada T, Gupta AK, Khokhar S, Vanathi M (2011). Ahmed glaucoma valve in post-penetrating-keratoplasty glaucoma: a critically evaluated prospective clinical study.. Indian J Ophthalmol.

[14] Papadaki TG, Zacharopoulos IP, Pasquale LR, Christen WB, Netland PA, Foster CS (2007). Long-term results of Ahmed glaucoma valve implantation for uveitic glaucoma.. Am J Ophthalmol.

[15] Shah MR, Khandekar RB, Zutshi R, Mahrooqi R (2013). Short term outcome of Ahmed glaucoma valve implantation in management of refractory glaucoma in a tertiary hospital in Oman.. Oman J Ophthalmol.

[16] Zhang HT, Yang YX, Xu YY, Yang RM, Wang BJ, Hu JX (2014). Intravitreal bevacizumab and Ahmed glaucoma valve implantation in patients with neovascular glaucoma.. Int J Ophthalmol.

[17] Spaeth GL (1990). Ophthalmic surgery: principles and practice..

[18] Chen PP, Yamamoto T, Sawada A, Parrish RK 2nd, Kitazawa Y (1997). Use of antifibrosis agents and glaucoma drainage devices in the American and Japanese Glaucoma Societies.. J Glaucoma.

[19] Desai MA, Gedde SJ, Feuer WJ, Shi W, Chen PP, Parrish RK 2nd (2011). Practice preferences for glaucoma surgery: a survey of the American glaucoma society in 2008. Ophthalmic Surg Lasers Imaging.

[20] Joshi AB, Parrish RK 2nd, Feuer WF (2005). 2002 survey of the American Glaucoma Society: practice preferences for glaucoma surgery and antifibrotic use.. J Glaucoma.

[21] Gedde SJ, Herndon LW, Brandt JD, Budenz DL, Feuer WJ, Schiffman JC (2007). Surgical complications in the Tube Versus Trabeculectomy Study during the first year of follow-up.. Am J Ophthalmol.

[22] Gedde SJ, Schiffman JC, Feuer WJ, Herndon LW, Brandt JD, Budenz DL (2007). Treatment outcomes in the tube versus trabeculectomy study after one year of follow-up.. Am J Ophthalmol.

[23] HaiBo T, Xin K, ShiHeng L, Lin L (2015). Comparison of Ahmed glaucoma valve implantation and trabeculectomy for glaucoma: a systematic review and meta-analysis.. PloS One.

[24] Nouri-Mahdavi K, Caprioli J (2003). Evaluation of the hypertensive phase after insertion of the Ahmed Glaucoma Valve.. Am J Ophthalmol.

[25] Tran DH, Souza C, Ang MJ, Loman J, Law SK, Coleman AL, Caprioli J (2009). Comparison of long-term surgical success of Ahmed Valve implant versus trabeculectomy in open-angle glaucoma.. Br J Ophthalmol.

[26] Tsai JC, Johnson CC, Kammer JA, Dietrich MS (2006). The Ahmed shunt versus the Baerveldt shunt for refractory glaucoma II: longer-term outcomes from a single surgeon.. Ophthalmology.

[27] Wilson MR, Mendis U, Paliwal A, Haynatzka V (2003). Long-term follow-up of primary glaucoma surgery with Ahmed glaucoma valve implant versus trabeculectomy.. Am J Ophthalmol.

[28] Lee TY, Lee JH, Cha SC (2008). Trabeculectomy with mitomycin C versus Ahmed valve implantation in pseudophakic glauco-matous eyes.. J Korean Ophthalmol Soc.

[29] Pakravan M, Homayoon N, Shahin Y, Ali Reza BR (2007). Trabeculectomy with mitomycin C versus Ahmed glaucoma implant with mitomycin C for treatment of pediatric aphakic glaucoma.. J Glaucoma.

[30] Shen CC, Salim S, Du H, Netland PA (2011). Trabeculectomy versus Ahmed Glaucoma Valve implantation in neovascular glaucoma.. Clin Ophthalmol.

[31] Budenz DL, Barton K, Feuer WJ, Schiffman J, Costa VP, Godfrey DG, Buys YM (2011). Ahmed Baerveldt Comparison Study Group. Treatment outcomes in the Ahmed Baerveldt Comparison Study after 1 year of follow-up.. Ophthalmology.

[32] Ishida K, Netland PA, Costa VP, Shiroma L, Khan B, Ahmed II (2006). Comparison of polypropylene and silicone Ahmed Glaucoma Valves.. Ophthalmology.

[33] Devgan U, Yu F, Kim E, Coleman AL (2000). Surgical undertreat-ment of glaucoma in black beneficiaries of Medicare.. Arch Ophthalmol.

[34] Ederer F, Gaasterland DA, Dally LG, Kim J, Van Veldhuisen PC, Blackwell B, Prum B, Shafranov G, Allen RC, Beck A (2004). The Advanced Glaucoma Intervention Study (AGIS): 13. Comparison of treatment outcomes within race: 10-year results.. Ophthalmology.

[35] Mermoud A, Salmon JF, Murray AD (1993). Trabeculectomy with mitomycin C for refractory glaucoma in blacks.. Am J Ophthalmol.

[36] Morris DA, Peracha MO, Shin DH, Kim C, Cha SC, Kim YY (1999). Risk factors for early filtration failure requiring suture release after primary glaucoma triple procedure with adjunctive mitomycin.. Arch Ophthalmol.

[37] Ishida K, Netland PA (2006). Ahmed Glaucoma Valve implantation in African American and white patients.. Arch Ophthalmol.

[38] Abe RY, Tavares CM, Schimiti RB, Vasconcellos JP, Costa VP (2015). Ahmed glaucoma valve implantation for refractory glaucoma in a tertiary hospital in Brazil.. J Ophthalmol.

[39] Eibschitz-Tsimhoni M, Schertzer RM, Musch DC, Moroi SE (2005). Incidence and management of encapsulated cysts following Ahmed glaucoma valve insertion.. J Glaucoma.

[40] Law SK, Kornmann HL, Giaconi JA, Kwong A, Tran E, Caprioli J (2016). Early aqueous suppressant therapy on hypertensive phase following glaucoma drainage device procedure: a randomized prospective trial.. J Glaucoma.

[41] Pakravan M, Rad SS, Yazdani S, Ghahari E, Yaseri M (2014). Effect of early treatment with aqueous suppressants on Ahmed glaucoma valve implantation outcomes.. Ophthalmology.

